# Cup Blocks the Precocious Activation of the Orb Autoregulatory Loop

**DOI:** 10.1371/journal.pone.0028261

**Published:** 2011-12-02

**Authors:** Li Chin Wong, Paul Schedl

**Affiliations:** Department of Molecular Biology, Princeton University, Princeton, New Jersey, United States of America; Stockholm University, Sweden

## Abstract

Translational regulation of localized mRNAs is essential for patterning and axes determination in many organisms. In the *Drosophila* ovary, the germline-specific Orb protein mediates the translational activation of a variety of mRNAs localized in the oocyte. One of the Orb target mRNAs is *orb* itself, and this autoregulatory activity ensures that Orb proteins specifically accumulate in the developing oocyte. Orb is an RNA-binding protein and is a member of the cytoplasmic polyadenylation element binding (CPEB) protein family. We report here that Cup forms a complex *in vivo* with Orb. We also show that *cup* negatively regulates *orb* and is required to block the precocious activation of the *orb* positive autoregulatory loop. In *cup* mutant ovaries, high levels of Orb accumulate in the nurse cells, leading to what appears to be a failure in oocyte specification as a number of oocyte markers inappropriately accumulate in nurse cells. In addition, while *orb* mRNA is mislocalized and destabilized, a longer poly(A) tail is maintained than in wild type ovaries. Analysis of Orb phosphoisoforms reveals that loss of *cup* leads to the accumulation of hyperphosphorylated Orb, suggesting that an important function of *cup* in *orb*-dependent mRNA localization pathways is to impede Orb activation.

## Introduction

In eukaryotic cells, cytoplasmic polyadenylation is used to activate the on-site translation of localized mRNAs. Polyadenylation is thought to depend upon two key elements in the 3′ UTR of the mRNA. The first is the AAUAAA motif, which is bound by the cleavage and polyadenylation specificity factor (CPSF) while the second is the U-rich cytoplasmic polyadenylation element (CPE) which is bound by the cytoplasmic polyadenylation element binding protein (CPEB) [1], [2]. The evolutionarily conserved CPEBs are RNA-recognition motif (RRM)-type RNA-binding proteins and they have been found in species ranging from nematodes to humans. One of the founding members of the CPEB family is the *Drosophila* germline-specific protein Orb [3]. Orb plays a critical role in the development of the female germline and is required for mRNA localization and translational regulation throughout much of oogenesis [4], [5].

Phenotypic analysis of strong *orb* alleles indicates that the formation of the 16-cell cyst, the specification of the oocyte and the proper expression of the TGF-α signaling molecule Gurken (Grk) at the posterior of pre-vitellogenic egg chambers requires *orb* activity [5]–[7]. In the hypomorphic allele *orb^mel^*, these early steps in oogenesis appear normal; however, the specification of both the anterior-posterior (AP) and dorsal ventral (DV) polarity axes in vitellogenic egg chambers is disrupted. Orb protein is thought to function in AP axis specification by binding to *oskar* (*osk*) mRNA after it is localized to the posterior pole of the oocyte and activating its translation by a mechanism involving polyadenylation [4], [8], [9]. In the DV polarity pathway, Orb is required for the localized translation of *grk* mRNA at the dorsal-anterior corner of the oocyte. In *orb^mel^* ovaries, *grk* mRNA is mislocalized and little or no Grk protein is produced. Like other mutations that disrupt *grk* signaling, *orb^mel^* eggs have ventralized chorions that either have fused or lack dorsal respiratory appendages [4], [7].

Another Orb regulatory target is its own mRNA. Orb is required to localize *orb* mRNAs to sites in the oocyte cortex and to promote their on-site translation. As is the case for *osk* mRNA, it is thought to act by binding to target sequences in the *orb* 3′ UTR and activating polyadenylation [10]. This positive autoregulatory activity ensures that high levels of Orb specifically accumulate in the oocyte, which is the compartment that requires *orb* activity. Since *orb* mRNA is synthesized in the nurse cells, and must be transported through the nurse cells into the oocyte and then localized within the oocyte to the cortex, there must be mechanisms in place that prevent the precocious activation of the *orb* positive autoregulatory loop. Previous studies have shown that the *Drosophila* Fragile X mental retardation protein dFMR1 downregulates *orb* mRNA translation in nurse cells [11]; however, the effects of *dfmr1* mutations on Orb protein expression, and on oogenesis in general, are relatively modest and it seems likely that other factors may play more central roles in blocking the premature activation of the *orb* positive autoregulatory loop.

One candidate for a gene that prevents the precocious activation of the *orb* positive autoregulatory loop is *fs(2)cup* (*cup*). *cup* was discovered in a screen for female sterile mutations [12]. Strong loss-of-function Class I alleles arrest oogenesis prior to the onset of vitellogenesis and they accumulate many small, round and abnormal-looking egg chambers [13]. Moderate Class II alleles progress farther; the egg chambers appear to take up yolk and have a more elongated shape. Oogenesis in Class III alleles is relatively normal up until stage 9–10 when the oocyte stops growing and this gives rise to cup-like chorions. The Cup protein has been shown to function as a translational repressor of several mRNAs including one of the known *orb* targets, *osk* [14], [15]. While the mechanism of repression is not fully understood, Cup has been shown to interact directly with three other translation factors, the cap-binding initiation factor eIF4e, and the RRM-type RNA-binding proteins Bruno and Smaug [15]–[17].

These interactions involve different domains of the Cup protein. For example, Cup-eIF4E interactions are mediated by a canonical and a non-canonical eIF4E binding motif in the Cup N-terminus, while the C-terminal end of the Cup protein mediates interactions with Bruno [15]–[17]. Though Cup has no known RNA binding activity, protein-protein interactions with Bruno (or Smaug) would function to recruit Cup to mRNAs like *osk* and *orb* that contain sequence motifs recognized by the Bruno protein. Cup is thought to inhibit the translation of these mRNAs by binding to eIF4E and sequestering it from interacting with eIF4G [15]–[17]. This prevents the assembly of the eIF4F initiation complex (consisting of eIF4A, eIF4E and eIF4G) and the loading of the 40S ribosomal subunit at the 5′ end of the mRNA [16]–[18]. Consistent with this model, the translation of *osk* mRNA is prematurely upregulated in several *cup* hypomorphic mutant combinations and Osk protein can be detected in stage 6–7 egg chambers [14], [15]. Moreover, in older mutant chambers, translation appears to be activated at the anterior of the oocyte instead of the posterior. Further supporting this model, premature activation of *osk* mRNA translation is also observed in a *cup* mutant, *cup^Δ212^*, which lacks the canonical high affinity eIF4E binding motif [15]. Interestingly, however, though the interaction of the mutant Cup^Δ212^ protein with eIF4E is expected to be compromised, it is only a very weak Class III allele. This observation suggests that the regulatory activities of *cup* during oogenesis are likely to include other functions besides sequestering the eIF4E translation factor.

In this paper, we present evidence that one of the other functions of the *cup* gene is to prevent the premature activation of the *orb* positive autoregulatory loop. In wild type ovaries, the *orb* autoregulatory loop is activated in the oocyte ensuring that high levels of Orb protein accumulate in the compartment where its activity is required. In contrast, in *cup* mutants the autoregulatory appears to be precociously activated and high levels of Orb protein accumulate in the nurse cells. Our data suggest that Cup employs at least two different though likely overlapping mechanisms to prevent the premature activation of the *orb* autoregulatory loop. The first is to limit the poly(A) tail length of *orb* mRNAs. In *cup* mutants *orb* mRNAs have longer poly(A) tails than in wild type. The second is to limit the accumulation of hyperphosphorylated (activated) Orb protein isoforms. In wild type ovaries, there are two Orb isoform populations, hypo- and hyperphosphorylated, that differ in the extent of phosphorylation and in their activity [19]. Most of the Orb protein in wild type ovaries is hypophosphorylated. In contrast, in *cup* ovaries there is a shift in the isoform distribution and most the Orb protein is hyperphosphorylated. In addition to these effects on *orb* expression and post-translational modifications, we find that *cup* is required for the proper localization and stability of *orb* mRNA.

## Results

### Cup associates with Orb *in vivo*

To identify components of the machinery that regulates Orb activity or localization, we searched for proteins that associate with Orb *in vivo*. For this purpose, we tested candidate proteins that were detected in a previous mass spectrometry analysis on Orb- immunoprecipitated wild type ovary extracts [19]. To enrich for proteins that are associated with Orb because they are in the same protein complexes rather than being linked together via an RNA bridge, we immunoprecipitated in the presence of RNase A. The proteins recovered from both the Orb and Dorsal immunoprecipitates were then analyzed by MudPIT, a mass spectrometry technology used for identifying proteins in complex mixtures ([19]; see also [11]). Altogether, ∼170 proteins were detected in Orb but not Dorsal immunoprecipitates. Proteins that were only found in Orb immunoprecipitates include over 30 ribosomal proteins, PABP, the *Drosophila* Gld2-homolog Wisp, five predicted RNA helicases, multiple RNA binding proteins, components of the siRNA machinery and proteins involved in decapping and RNA turnover like Me31B, Trailer Hitch, Not4, Enhancer of decapping and Bicaudal-C. There were also several proteins (e.g., Encore, Didum, Ovarian tumor and Oskar) implicated in mRNA localization in *Drosophila* ovaries. We also found Cup and one of its known partners, the initiation factor eIF4E [15].

**Figure 1:**
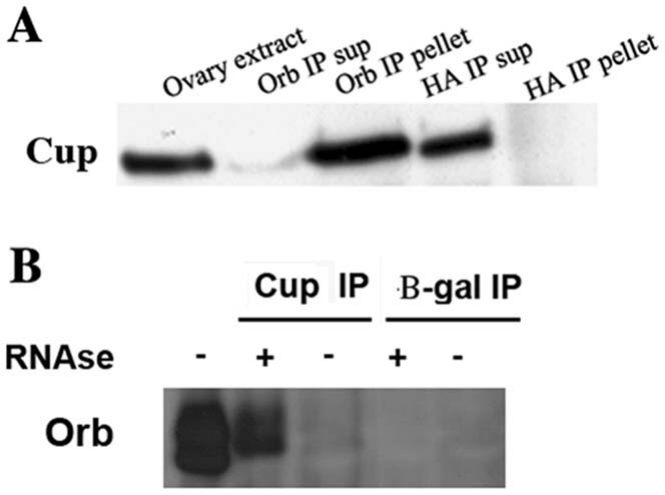
Cup associates with Orb *in vivo* and affects the phosphorylation status of Orb. Cup was initially identified as an Orb associated protein by analyzing Orb antibody immunoprecipitates from wild type ovaries using MudPIT mass spectrometry [19]. Western blots were used to confirm this association. (A): Immunoprecipitation of ovarian extracts with Orb antibody co-immunoprecipitates Cup protein. Cup does not co-immunoprecipitate with a negative control, HA antibody. The amount of ovary extract loaded in panel A represents 5% of the ovary extract used in the Orb and HA antibody immunoprecipitates. Half of the sample recovered in each immunoprecipitate was loaded on to the “pellet” lanes. For HA, approximately 2.5% of the sample was loaded into the “supernatant” lane. For the Orb “supernatant” lane a portion of the sample was lost during loading. (B): The converse immunoprecipitation, using antibody against Cup, pulled down both Orb isoform, whereas β-galactosidase antibody does not. The Cup-Orb complexes are RNA-independent as they are resistant to treatment with RNAse A. The amount of ovary extract loaded in the first lanes in each panel consist of 10% of the amount of ovary extracts used in each immunoprecipitation experiment loaded in subsequent lanes. The sample loaded onto the HA “supernatant” lane represents in panel A represents 2.5% of the supernatant from the Cup antibody immunoprecipitation precipitation.

To confirm the physical association between Cup and Orb, we immunoprecipitated ovarian extracts with either Cup or Orb antibodies and then analyzed the immunoprecipitates by Western blotting. Orb, but not HA antibody is able to pull-down Cup protein (Fig. 1A). The total amount of lysate, which was used in the pull-down, and then loaded in the IP lane, was approximately ten-fold greater than the lysate which was loaded in the extract lane. Thus, only about 10% of the Cup protein is pulled down by Orb antibody. While IP efficiency could account for this difference, another factor that is likely to be important is that Orb protein is largely restricted to the oocyte. By contrast, Keyes and Spradling [13] have shown that while Cup is somewhat enriched in the oocyte especially in early stages of oogenesis, there are nevertheless substantial amounts of Cup protein in nurse cells.

In the converse experiment, we found that Cup antibody co-immunoprecipitates Orb protein (Fig. 1B). This association also does not depend upon an RNA bridge as RNase A treatment does not disrupt the Cup-Orb association. In fact, the amount of Orb pulled down in Cup immunoprecipitates seems to be greater when RNAse is present during the immunoprecipitation, compared to when RNAse is not present. These findings may indicate that the epitopes recognized by the Cup antibodies are occluded in native Orb-Cup complexes that contain mRNAs (and in some cases polyribosomes; [19]). When these complexes are treated with RNase, they may rearrange or lose factors that prevent immunoprecipitation of Orb with Cup antibodies.

### *cup* negatively regulates *orb*

To determine if the physical association between *orb* and *cup* is functionally important, we tested for genetic interactions. *orb* is weakly haploinsufficient for its activity in the establishment of DV polarity in the developing egg chamber, and 5–10% of the eggs laid by females heterozygous for the null allele *orb^343^* have ventralized chorions due to a defect in the production of the Grk ligand. It is possible to exacerbate this haploinsufficiency by introducing a dominant negative transgene *HD19G*. *HD19G* expresses an hybrid mRNA that contains β-galactosidase protein coding sequence fused to the 3′ UTR of *orb* mRNA [10]. The *orb* 3′ UTR in the chimeric mRNA competes with the 3′ UTR of the endogenous *orb* mRNA for Orb protein binding. This interferes with *orb* autoregulation and downregulates Orb protein expression. When this transgene is combined with the *orb* null allele, *orb^343^*, 20–30% of the eggs laid by transgene-heterozygous mutant females are ventralized. The somewhat higher frequency of DV defects in the *HD19G orb^343^/+* background as compared to *orb^343^/+* is useful in that it allows a more reliable detection of mutations that dominantly suppress the DV polarity defects induced by compromising *orb* activity. It is expected that dominant suppressors will correspond to genes that function to repress or reduce *orb* activity and/or *grk* signaling. In contrast, mutations that dominantly enhance the *HD19G orb^343^/+* DV polarity defects are expected to correspond to genes that are required to promote *orb* activity and/or *grk* signaling.

We generated females *trans*-heterozygous for a recombinant *HD19G orb^343^* chromosome and various *cup* mutant alleles by crossing *HD19G orb^343^/TM3Ser* females and *cup/CyO* males to test for genetic interactions. Using this strategy, we analyzed 5 different *cup* alleles, *cup^1^*, *cup^3^*, *cup^6^*, *cup^8^*, and *cup^1355^* [12], [13]. *cup^3^*, *cup^6^* and *cup^8^* are strong loss-of-function mutations and these Class I alleles exhibit a pre-vitellogenesis oogenesis arrest. *cup^1^* is a Class II allele and ovarian development arrests around stage 9. Finally, *cup^1355^*, which has a P-element insertion in the 5′ UTR that downregulates its expression, is a Class III allele and arrests during vitellogenesis.

**Figure 2:**
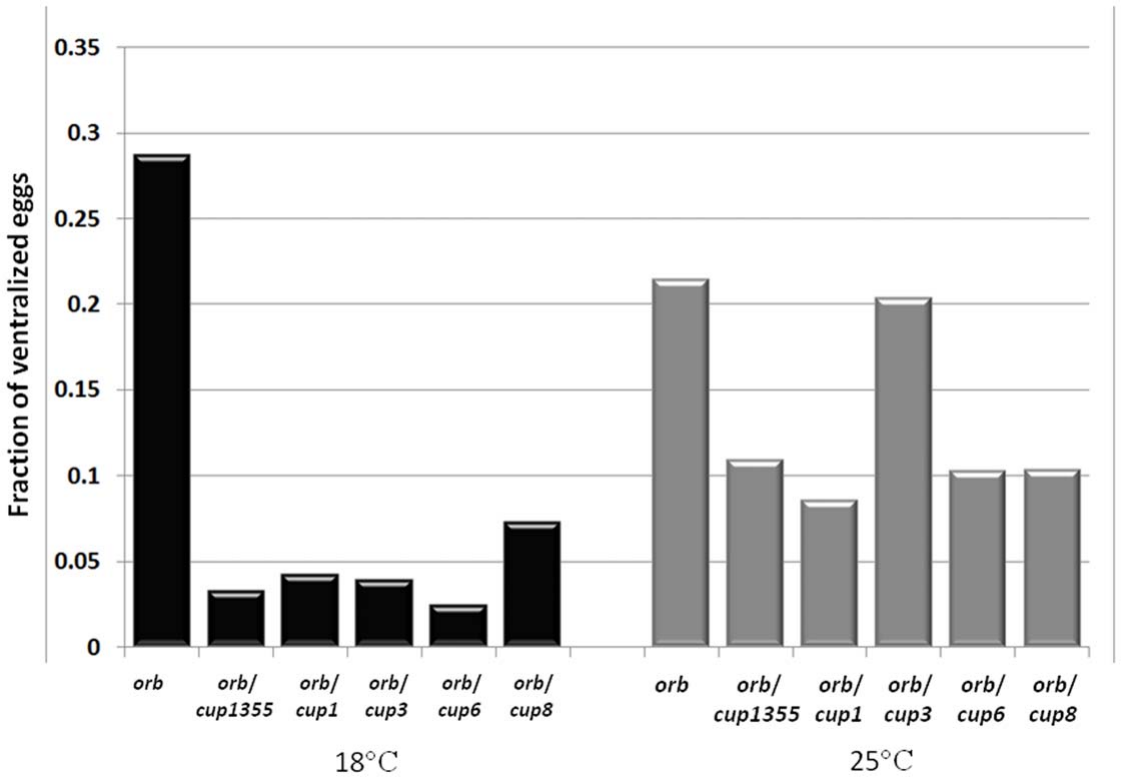
Cup negatively regulates *orb*. *orb* is weakly haploinsufficient for the specification of dorsal-ventral polarity and a small percentage of the eggs produced by *orb^343^/+* females are ventralized. This haploinsufficiency is enhanced when the *orb^343^/+* females also carry a copy of the *hsp83-LacZ orb* 3′ UTR transgene and, depending upon temperature and growth conditions, between 20%–30% of the eggs laid by *HD19G orb^343^/+* females are ventralized. To test for genetic interactions with *cup*, *HD19G orb^343^/TM3Ser* females (n = 10 in each cross) were mated with *cup/CyO* males. Five different *cup* alleles, *cup^1^*, *cup^3^*, *cup^6^*, *cup^8^* and *cup^1355^* were tested at 18°C and 25°C. When *trans*-heterozygous, all 5 alleles reduce the frequency of DV polarity defects in eggs produced by *HD19G orb^343^/+* females. The severity of this defect ranges from completely fused to missing dorsal appendages. Suppression is stronger at 18°C (black bars) than at 25°C (gray bars). The p-values (by Chi-squared test) for each experiments are as such: 18°C (*cup^1^*: 3.031E-141; *cup^3^*: 1.7326E-29; *cup^6^*: 7.6267E-36; *cup^8^*: 7.6611E-14; *cup^1355^*: 1.72342E-28) and 25°C: (*cup^1^*: 5.428E-118; *cup^3^*: 0.608012094; *cup^6^*: 4.5796E-09; *cup^8^*: 0.000309325; *cup^1355^*: 4.52924E-07).

As illustrated in Fig. 2 (and Table S1), we found that the DV polarity defects in eggs produced by *HD19G orb^343^/+* females can be suppressed by reducing *cup* activity. At 25°C, the frequency of DV defects in eggs from *HD19G orb^343^/+*; *cup^−^/+* females of severe alleles is, with one exception (*cup^3^*), about half that seen for *HD19G orb^343^/+* females. The suppression is even more pronounced at 18°C. At this temperature, close to 30% of the eggs laid by *HD19G orb^343^/+* females are ventralized while in the *cup trans*-heterozygotes the frequency of egg shell polarity defects drops to less than 10%.

In a separate experiment (not shown) we also tested the very weak Class III allele, *cup^Δ212^*, which lacks the canonical eIF4E interaction domain. Unlike stronger *cup* alleles, which arrest oogenesis and can substantially alter the pattern of *orb* expression (see below), oogenesis is comparatively normal in *cup^Δ212^* and defective eggs are produced [15]. This allele had, at most, only a modest effect on the frequency of ventralized eggs produced by *HD19G orb^343^/+* females. Thus, the canonical eIF4E interaction domain is unlikely to be critical for genetic interactions between *cup* and *orb* at least in the DV polarity pathway.

### Orb protein levels are altered in *cup* ovaries

**Figure 3:**
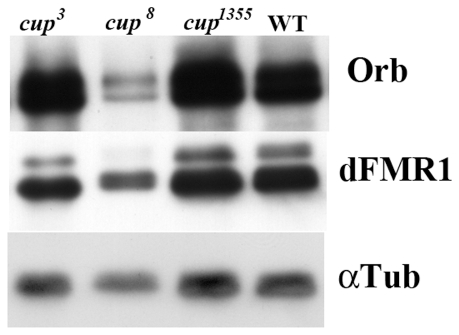
Orb protein levels are altered in *cup* mutant ovaries. A Western blot of ovary extracts from wild type and *cup* mutants as indicated was probed sequentially with Orb, dFMR1 and α-tubulin antibodies. Orb protein levels are reduced in *cup^8^* ovaries compared to wild type. The ratio of *cup^8^/*WT is 0.56 using dFMR1 as a loading control and 0.34 using α-tubulin as a loading control. In contrast, Orb levels are elevated in *cup^3^* and *cup^1355^* ovaries. For *cup^3^*, the level of Orb protein is elevated 1.8-fold using dFMR1 as a loading control and 1.9 using α-tubulin as the loading control. For *cup^1355^*, the level of Orb protein is elevated 1.6-fold using dFMR1 as a loading control, and 2.2-fold using α-tubulin as the loading control. Even larger increases were observed in another experiment: *cup^3^*: 3.0-fold using dFMR1 as the loading control and 2.25-fold using α-tubulin; *cup^1355^*: 2.9-fold using dFMR1 as the loading control and 2.8-fold using α-tubulin as the loading control.

The genetic interactions described above suggest that in addition to their physical association, there may also be a functional relationship between *cup* and *orb*. Moreover, given what is already known about the regulatory activities of the Cup protein in other contexts [14]–[16], one plausible explanation for the genetic interactions is that *cup* negatively regulates *orb* expression and/or activity. In this case, we would expect to find that Orb protein levels are increased in *cup* mutant ovaries. To test this prediction, we probed Western blots of ovary extracts prepared from female homozygous mutants for three different *cup* alleles, *cup^8^*, *cup^3^* and *cup^1355^* with Orb antibodies. We anticipated that Orb levels would be elevated in the mutants if *cup* functions as a “general” negative regulator of *orb* activity (because of Orb autoregulation), and that in this case the effects on Orb accumulation should be proportional to the strength of the *cup* allele. However, the results were anomalous (Fig. 3). Instead of having the highest levels, the amount of Orb protein in the strongest mutant, *cup* ^8^, was actually less than in wild type. In contrast, the amount of Orb protein in the weaker Class I allele *cup^3^*, and in the Class III allele, *cup^1355^*, was greater than wild type. The reason for the unusual effects of the *cup* mutations became apparent when we examined the pattern of Orb protein accumulation in ovaries from the different mutants using confocal microscopy.

### Orb is mislocalized and misexpressed in *cup* ovaries

**Figure 4:**
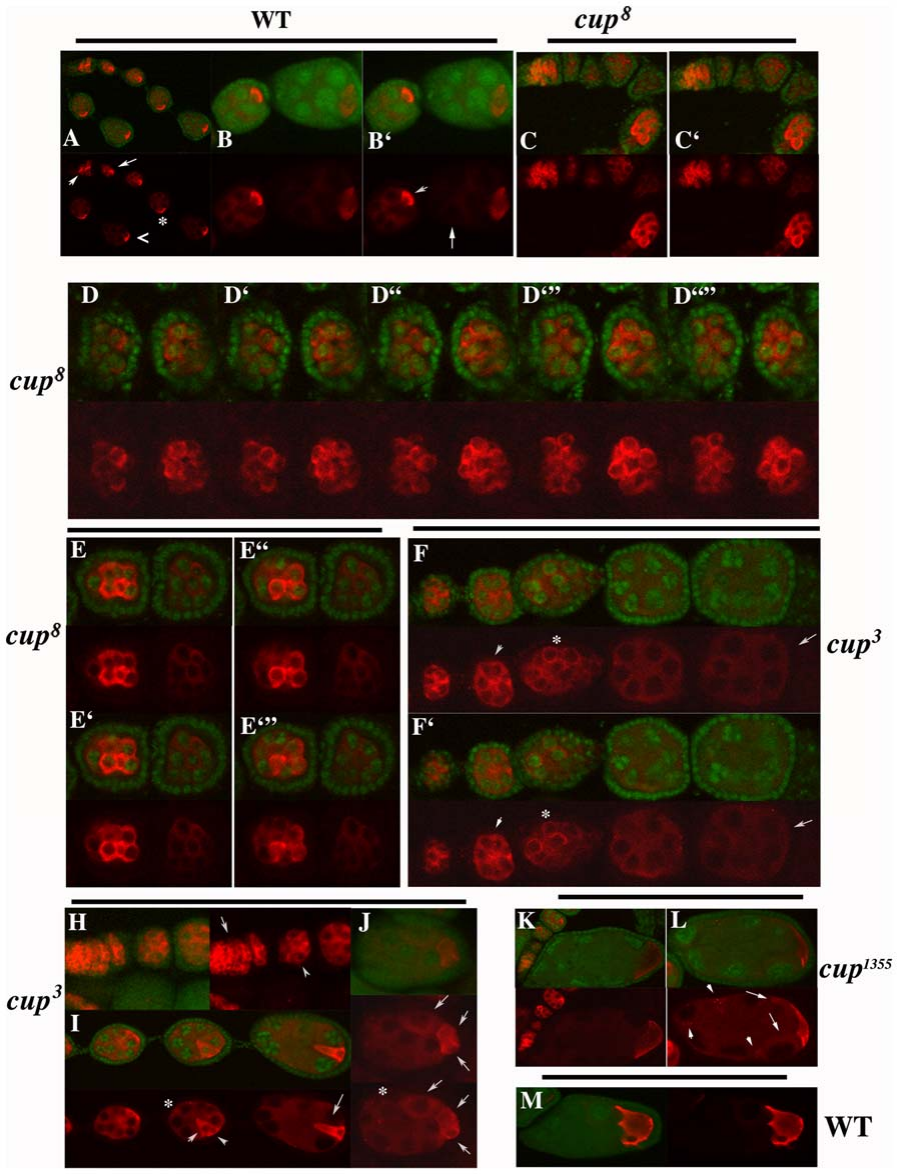
Orb is misexpressed in *cup* ovaries. Green: Nucleic acids. Red: Orb. Panels A–B′: In wild type, Orb is first expressed during the formation of the 16-cell cyst (arrowhead in A) and though it is present in most cells in the cyst, it is greatly enriched in the presumptive oocyte. The localization pattern is refined during early pre-vitellogenic stages (arrow in A); Orb becomes concentrated at the posterior of the oocyte (>and * in A, arrowhead in B′), while only low levels of protein are detected in the nurse cells closest to the oocyte (arrow in B′). Panels B and B′: successive confocal sections through the same two egg chambers. Note that there are high levels of Orb protein in all of the germ cells in these two chambers. Panels C, D and E: Chambers from *cup^8^* ovaries. In each case several focal planes are shown and are designated as ‘. In *cup^8^* 16-cell cysts Orb is present at high levels in most of the cells (C, C′). Unlike wild type the Orb localization pattern does not refine during the early pre-vitellogenic stages and high levels of Orb persist in most of the cells in the chamber. The distribution of Orb in two “early” *cup^8^* chambers is shown in panels D-D″″. Note that many cells in these two chambers have high levels of Orb protein. Two other *cup^8^* chambers are shown in panels E-E″. In the chamber on the left Orb is clearly concentrated in a subset of the cells. In the chamber on the right the level of Orb protein is low. A marked reduction in the level of Orb protein is typically observed in older *cup^8^* chambers. Panels F–J show the pattern of Orb accumulation in *cup^3^* chambers. Like *cup^8^*, chambers that have high levels of Orb in most germ cells are often observed in the *cup^3^* mutant (arrowhead in F, F′ and H). Orb also accumulates in rings around the nurse cell nuclei (* in F, F′) or in clumps in the nurse cell cytoplasm (* in I and J). In some *cup^3^* chambers Orb concentrates in several cells near the posterior (arrow heads in I, arrows in J) while in other chambers the oocyte has an abnormal elongated shape (arrow in I). Panels K and L show *cup^1355^* chambers while panel M shows a wild stage 7–8 chamber. Orb accumulation during the early stages of oogenesis usually appears normal in *cup^1355^* (panel K); however in older chambers, high than normal levels Orb protein are seen in the nurse cells (arrows in L) and there are often clumps of Orb protein (arrowheads in L). In older wild type chambers (panel M) Orb is localized to the oocyte cortex, while there is little Orb protein in the nurse cells.

In wild type ovaries, Orb protein can first be readily detected in the germarium in newly formed 16-cell cysts. Though Orb is found in all 16 cells, it preferentially accumulates in one or only a small subset of these cells (arrowhead in Fig. 4A; see also [5]). The pattern of Orb protein localization then begins to refine and by the time the cells in the cyst rearrange and the oocyte moves to the posterior pole of the egg chamber, much of the Orb protein in the chamber is concentrated in the oocyte (arrow in Fig. 4A) while the nurse cells have considerably lower levels of Orb protein. Often a gradient of protein can be seen with the highest levels of Orb in the nurse cells closest to the oocyte. In slightly older chambers (* and<in Fig. 4A and arrow Fig. 4B′), Orb protein localizes to the posterior pole of the oocyte, while there are only very low levels of protein in the nurse cells.

A very different pattern of accumulation is evident in *cup^8^* ovaries. In the germarium, Orb does not concentrate into a single cell. Instead, high levels of protein are found in nearly every cell in the 16-cell cysts (Fig. 4C). High levels of largely unlocalized Orb protein typically persist in the *cup^8^* chambers after they exit the germarium and begin to “mature”. This can be seen in the pair of chambers shown in Fig. 4D. Most cells in these two chambers have high levels of Orb and it is not possible to determine which cell is the “oocyte” based on the Orb localization pattern. In other mutant chambers (see Fig. 4E), Orb becomes concentrated in 2–4 cells; however, even in these chambers it is often difficult to identify the oocyte. There are also chambers that appear (based on the concentration of Orb in a single cell) to have a properly “determined” oocyte; however, instead of being located at the posterior of the chamber, it is in the center or off to the side (Fig. S1). These findings argue that there are abnormalities in oocyte specification and/or maintenance of oocyte identity in *cup^8^* ovaries. Consistent with this conclusion, we find that another marker of oocyte identity, Encore [20], is also not properly localized in many *cup^8^* egg chambers. Figure S1 shows *cup^8^* chambers in which both Encore and Orb are enriched in several cells instead of a single cell.

While Orb protein levels in the germarium and in most early chambers appear to be considerably higher than in wild type (judging from the relative intensity of staining in wild type and *cup* chambers and from the fact that most of the germ cells in mutant chambers have substantial amounts of Orb), these high levels of protein do not persist. Instead, we find that Orb levels begin to drop as the chambers age and in older chambers (based on their location in the string of chambers) there is often little or no Orb present (chamber on right in Fig. 4E). However, the transition from high to little or no Orb does not always follow this time course, and we also observe ovarioles that have one or more older chambers with high levels of Orb and a younger chamber with little or no Orb. The presence of many chambers that have little or no Orb, likely explains why we observed a decrease instead of an increase in Orb protein levels in Western blots of *cup^8^* ovaries.

The oogenesis defects evident in the weaker Class I allele, *cup^3^*, are less severe than in *cup^8^*. Like *cup^8^*, the level of Orb protein in *cup^3^* chambers appears to be considerably higher than normal in the germarium (arrow in Fig. 4H) and in newly pinched-off chambers. Also like *cup^8^*, Orb protein is usually not restricted to a single cell (the presumptive “oocyte”), but instead is distributed at approximately the same level in many if not all of the germ cells in the chamber (arrowhead in Fig. 4F and H). In addition to excess Orb in nurse cell cytoplasm, there are often prominent rings of Orb protein around the nurse cell nuclei (see * in Fig. 4F). While similar rings of Orb can be detected in wild type ovaries [11], they are only observed in overexposed images. Interestingly, many proteins involved in mRNA transport including Cup [13] concentrate in rings around the nurse cell nuclei and these rings are thought to correspond to the sites of assembly of silenced mRNP complexes [21].

In *cup^8^* the level of Orb protein drops as the egg chambers age. While older chambers with reduced levels of Orb protein are seen in *cup^3^* ovarioles (arrows in Fig.4F, F′), we also find many older chambers that retain higher than normal levels of Orb protein. In some cases, most of the protein is concentrated in a single cell, which presumably corresponds to the oocyte. The presumptive oocyte in Fig. 4I (see arrow) has an unusual triangular shape. In other chambers, several cells located near the posterior have high levels of Orb (see arrowheads in 4I and arrows in 4J). In these chambers the overall level of Orb protein in the nurse cells also appears to be higher than in wild type. The presence of many older chambers in which Orb levels remain elevated likely explains why much greater amounts of Orb are detected in Western blots of *cup^3^* ovaries than in *cup^8^* ovaries.

While the oocyte appears to be mis-specified in *cup^8^* or (to a lesser extent) *cup^3^* chambers, this problem is usually not observed in the Class III *cup^1355^* mutant where the pattern of Orb accumulation appears to be comparatively normal in early stages 1–5 *cup^1355^* chambers (Fig. 4K). However, defects become apparent around stages 6–7. The oocyte in these chambers can have an abnormal shape and is often smaller than normal. Although Orb is enriched in the *cup^1355^* oocyte, the relative level of protein appears to be reduced compared to wild type oocytes. By contrast, the nurse cells of these older chambers have higher levels of Orb than wild type nurse cells (Fig. 4L) and Orb can be observed around the nurse cell nuclei or in clumps in the nurse cell cytoplasm (see arrows in L). Orb protein clumps are also seen in the nurse cell cytoplasm of *cup^3^* (see * in I). These findings indicate that *orb* mRNA is inappropriately translated in the nurse cells beginning during mid-oogenesis (stages 6–7) in *cup^1355^* mutant ovarioles. Interestingly, precocious expression of Osk is observed around the same time in other Class III *cup* mutants [15].

### *orb* mRNA is mislocalized in *cup* mutants

**Figure 5:**
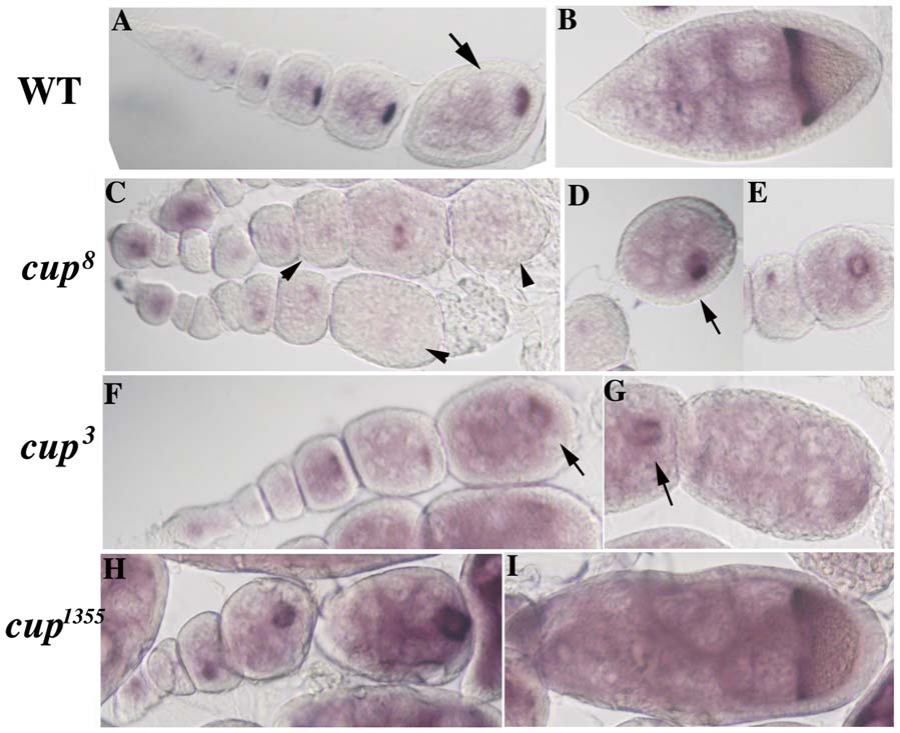
*orb* mRNA is mislocalized in *cup* mutants. In wild type, o*rb* mRNA can first be detected in the germarium (panel A). It appears concentrated in a single cell that is thought to correspond to the presumptive oocyte. During the previtellogenic stages (panel A), *orb* mRNA is localized at the posterior end of the oocyte, while there are only low levels in the nurse cell cytoplasm. After the onset of vitellogenesis (panel B), *orb* mRNA accumulates along the anterior margin of the oocyte. In many *cup^8^* chambers *orb* mRNA is not properly localized and reduced in level (arrowheads in C). Sometimes a single cell can be seen which has high levels of *orb* mRNA (D & E); however, this cell is not always positioned correctly (arrow in D). Mislocalized *orb* mRNA is also seen in *cup^3^*. In the examples shown in F and G the level of *orb* mRNA in the nurse cells is high, while the level in the presumptive oocyte is relatively low (compare levels of *orb* mRNA in nurse cells and oocyte in the wild type (arrow in panel A) and mutant (arrow in F and G) chambers). The pattern of *orb* mRNA accumulation in *cup^1355^* chambers (panels H and I) more closely resembles wild type; however, in older chambers the mRNA is not properly localized to the anterior margin and higher than normal levels are seen in the nurse cells (panel I).

To further investigate the effects of *cup* mutations on *orb*, we examined the pattern of *orb* mRNA accumulation in *cup* ovaries. In wild type ovaries, *orb* mRNA localizes preferentially to the oocyte soon after the 16-cell cyst is formed [3]. In pre-vitellogenic stages, most of the message is concentrated at the posterior pole of the oocyte, while after the onset of vitellogenesis, the message re-localizes along the anterior margin (Fig. 5A and 5B). In both *cup^8^* and *cup^3^* ovaries, *orb* message is distributed more or less uniformly in the germ cells of the egg chamber; however, many of these chambers have a single cell which has somewhat higher levels of mRNA (arrows in Fig. 5D, 5F and 5G) and this cell would presumably correspond to the “oocyte.” While this presumptive oocyte is usually near the posterior of the chamber in *cup^3^*, it can be in the center or off to one side in *cup^8^* chambers (panel D). Besides mislocalized mRNA, there are also chambers that have little or no *orb* message (see arrowheads in panel C). As was seen for Orb protein, the distribution of *orb* mRNA in *cup^1355^* more closely resembles wild type than either of the stronger alleles (panel H). However, even in this mutant the relative proportion of message in the nurse cells appears higher than in wild type chambers of similar stages (panel I).

These findings indicate that the *cup* activity is required not only to regulate the translation of *orb* mRNA but also for its proper localization. In addition, since many chambers (especially in the stronger alleles) have little *orb* mRNA, it would appear that *cup* is required either for the synthesis of *orb* mRNA, or to stabilize its accumulation. The latter possibility is supported by recent studies using a tissue culture system by Igreja and Izaurralde [22] who showed that Cup stabilizes reporter mRNAs when tethered to them. To determine if *cup* activity is also required for the normal accumulation of *orb* mRNA, we used quantitative real-time PCR. Compared to a normalized wild type value of 1.0 (for pre-vitellogenic egg chambers) the level of *orb* mRNA in *cup^8^*, *cup^3^* and *cup^1355^* mutant ovaries is 0.28, 0.32 and 0.32 respectively (see Experimental Procedures). These results would be consistent with the tissue culture experiments and would argue that *cup* is also required for *orb* mRNA stability.

### *osk* mRNA levels are also decreased in *cup* mutants

**Figure 6:**
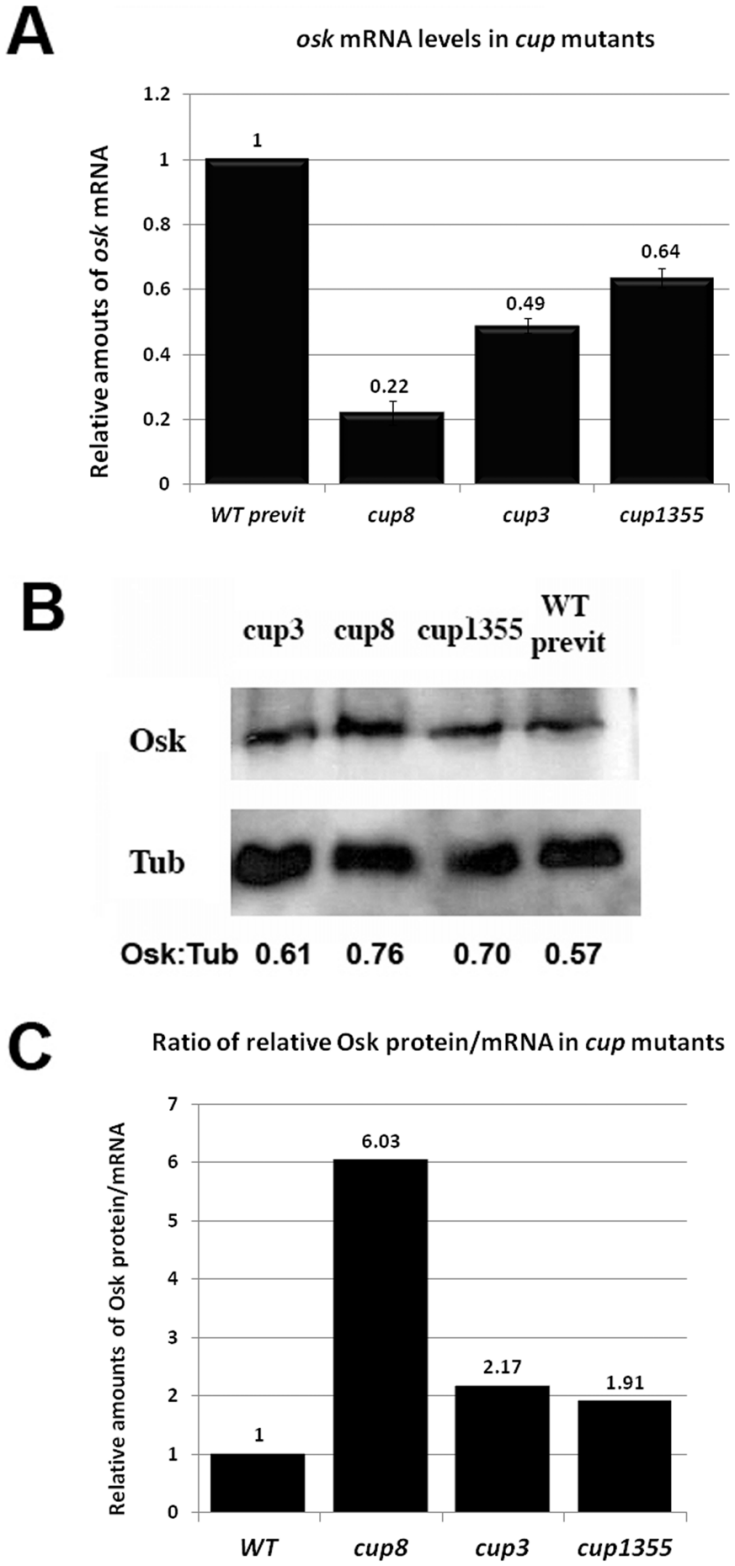
*osk* mRNA is reduced in *cup* mutants while Osk protein levels remain the same as in wild type. (A) *osk* mRNA levels in pre-vitellogenic wild type ovaries and in ovaries from the three *cup* alleles, *cup^8^*, *cup^3^* and *cup^1355^*, were measured by quantitative real time RT-PCR using *actin* mRNA as the control. *osk* mRNA levels in the mutant ovaries were found to be decreased compared to wild type levels. The largest reduction is seen *cup^8^*, where the level of *osk* mRNA is only about 20% that in previtellogenic wild type ovaries. In the less severe alleles, *osk* mRNA levels are 50% (*cup^3^*) and 70% (*cup^1355^*) of wild type previtellogenic levels. The amount of *osk* mRNA normalized to *actin* mRNA in pre-vitellogenic chambers is essentially the same (98%) as it is in ovaries from aged wild type females. (B) The effects of *cup* mutations on the expression of Osk protein. The relative amount of Osk protein in wild type and *cup* mutant ovaries was estimated by comparing the levels of Osk and α-tubulin in pre -vitellogenic wild type ovaries and in ovaries from the three *cup* mutants, *cup^8^*, *cup^3^* and *cup^1355^*. In all three cases the relative amount of Osk protein was marginally greater than the amount in wild type ovaries. (C) Panel C shows the ratio of Osk protein to *osk* mRNA levels. Assuming that the rate of Osk turnover in wild type is equivalent to that in the *cup* mutants, more Osk protein must produced on average from each *osk* mRNA in the *cup* mutants than in wild type.

Previous studies have shown that, like *orb* mRNA, one of the other known Orb regulatory targets, *osk* mRNA, is mislocalized and prematurely translated in *cup* ovaries [14], [15]. We wondered whether *osk* mRNA is also reduced in level when *cup* activity is compromised. To address this question, we used quantitative RT-PCR to estimate the relative abundance of *osk* mRNA in the three *cup* mutants. Figure 6A shows that there is less *osk* mRNA in the *cup* mutants than in wild type and that the extent of the reduction roughly corresponds to the severity of the allele. For example, *cup^8^*, the most severe mutant had approximately 22% of the *osk* mRNA found in wild type ovaries. Since the amount of Osk protein detected in Western blots of extracts from the three *cup* mutants is close to that found in wild type (pre-vitellogenic) chambers (see Fig. 6B), this would imply that more Osk is being expressed from less mRNA in the *cup* mutants (Fig. 6C).

### *orb* poly(A) tails are elongated in *cup* mutants

**Figure 7:**
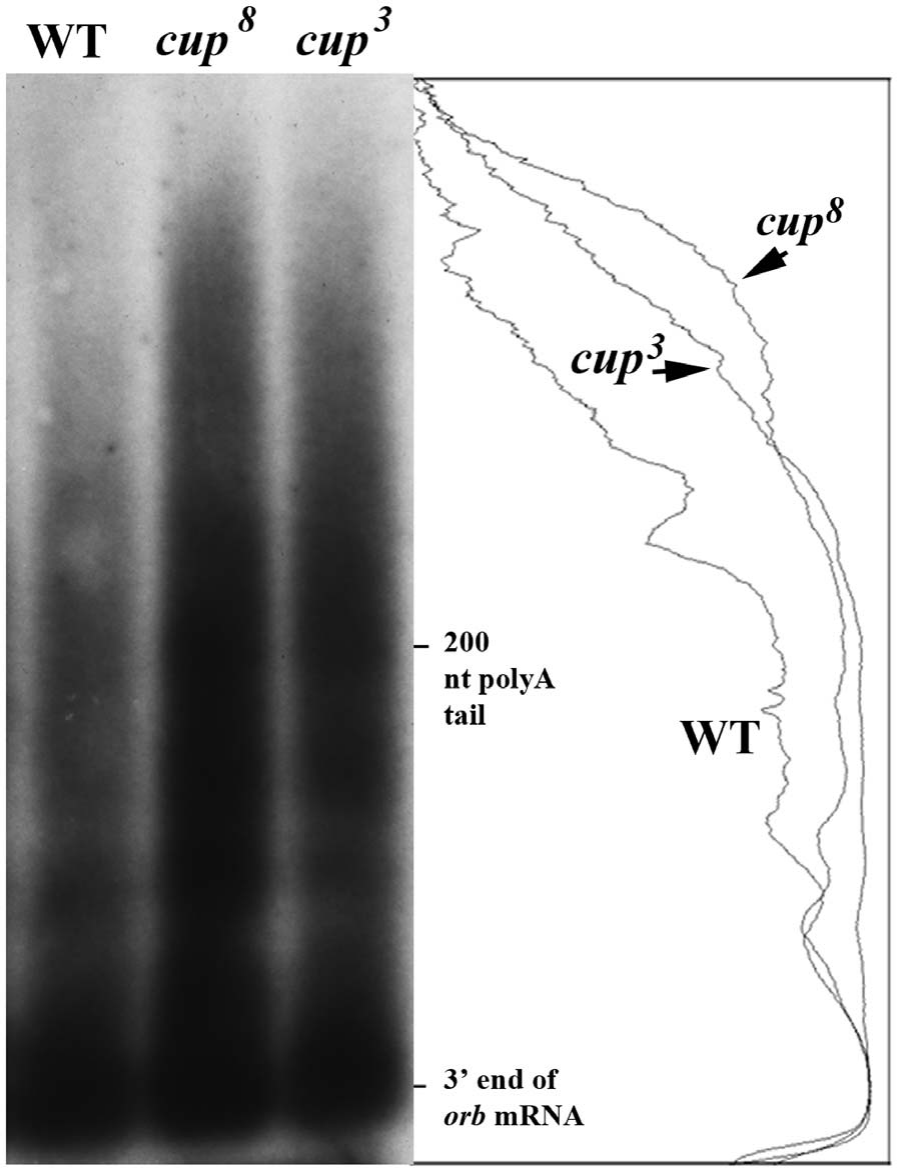
The poly(A) tails of *orb* in *cup^8^* and *cup^3^* mutants are elongated compared to wild type. Poly(A) tails of *orb* mRNA from wild type, *cup^8^* and *cup^3^* (as indicated) were analyzed using the ligation-mediated poly(A)-tail assay [24]. Anchor primers for reverse transcription and amplification from the poly(A) tail were as described in [24]. To increase specificity for the *orb* 3′ UTR we used a two step PCR amplification with a nested primer pair derived from the *orb* 3′UTR (F2:GATTGTCCGCTAAGCGTTTATCAGGA and F4:CCTTGTGAACATTAACGCGATG). The *orb* 3′UTR products from the 2^nd^ PCR amplification were analyzed on an agarose gel, and after blotting to nitrocellulose were detected by hybridizing the filter with a radioactive *orb* 3′UTR probe.

The findings described in the previous sections indicate that Orb protein is precociously and/or overexpressed in *cup* mutant ovaries. Since *orb* mRNA levels are also reduced, it would appear that they are translated more efficiently in the *cup* mutants. One mechanism that could account for a general increase in translational efficiency would be an increase in the average poly(A) tail length [23]. This idea is supported by the finding that Cup protein recruits components of the deadenylation machinery to target mRNAs [22]. According to this model, Cup would normally bind to newly synthesized *orb* mRNAs in nurse cells and promote their deadenylation. This would ensure that *orb* mRNAs remained repressed until they are localized in the oocyte. In *cup* mutants, *orb* mRNAs would have elongated poly(A) tails and this would increase their translational efficiency. A prediction of this model is that the poly(A) tails of *orb* mRNAs will be elongated in *cup* mutant ovaries. To determine whether this is true, we measured the lengths of *orb* mRNA poly(A) tails in wild type, *cup^8^* and *cup^3^* ovaries using the ligation-mediated poly(A)-tail assay [24]. We find that there is a shift in the distribution of *orb* mRNA poly(A) tails towards longer tails in both *cup^8^* and *cup^3^* ovaries (Fig. 7). A smaller shift towards longer poly(A) tails was also observed for *cup^1335^* using the anchored poly(A)-tail assay (Fig. S2) [24].

### Hyperphosphorylated Orb accumulates in *cup* ovaries

The findings described above indicate that *orb* mRNAs in *cup* ovaries have elongated poly(A) tails. While this would be expected to increase their translational efficiency [23], it is not clear that this would be sufficient in itself to account for the precocious expression of Orb protein in nurse cells, especially like that seen in the two stronger *cup* alleles. The abnormally high levels of Orb protein seen in the nurse cells suggests that the *orb* autoregulatory loop is also prematurely activated in the *cup* mutants. We've previously shown that *orb* activity is regulated, at least in part, by phosphorylation and that phosphorylation depends upon Casein Kinase II (CK2) [19]. In wild type ovaries there are two populations of Orb protein, the hypophosphorylated and hyperphosphorylated populations, both of which consist of several distinct phosphoisoforms. In females that are partially compromised for *ck2* activity, there is a shift in the *orb* phosphoisoform profile towards the hypophosphorylated isoforms. Correlated with this shift, we find that *orb* functioning in both autoregulation and in *grk* signaling is disrupted. The two phosphoisoform populations are also found in quite distinct protein complexes that likely to differ in their activities. The hypophosphorylated proteins are in slowly sedimenting complexes and are associated with the translational Bruno repressor [18]. Bruno repressor also interacts directly with Cup and this interaction is important for Cup-mediated repression [15]. While the complexes containing hypophosphorylated isoforms appear to be involved in repression, the hyperphosphorylated isoforms are associated with complexes likely involved in translational activation. The hyperphosphorylated isoforms co-sediment with poly-ribosomes and are associated with the polyA polymerase Wisp which is thought to promote mRNA activation [25], [26]. Hence, one mechanism that could lead to the inappropriate activation of the *orb* autoregulatory loop would be to upregulate Orb phosphorylation.

**Figure 8:**
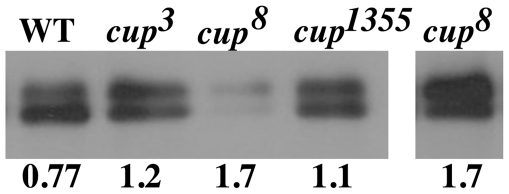
The ratio of the upper and lower Orb isoforms is altered in *cup* ovaries. In wild type, the lower isoform is typically most abundant, while in *cup* mutant ovaries the level of the upper isoform is increased. The ratio of upper to lower phosphoisoforms in the WT is 0.75. In other experiments (n = 6) the range for WT was 0.74–0.84 with an average value of 0.77. In the experiment shown here, the ratio for the *cup* mutants is increased to 1.3, 1.9 and 1.1 for *cup^3^*, *cup^8^* and *cup^1355^*, respectively. Two different exposures of the *cup^8^* lane are shown. The average of three experiments for *cup^3^* was 1.2, *cup^8^* was 1.7 and *cup^1355^* was 1.1. The average ratio is shown in the figure.

To test whether *cup* affects the Orb phosphorylation status, we separated the hypo- and hyperphosphorylated Orb isoforms in wild type and *cup* mutant ovaries on SDS-PAGE gels. Since *cup* ovaries arrest early in oogenesis, we compared the Orb protein in *cup* ovaries with wild type ovaries containing only pre-vitellogenic stages. As shown in Fig. 8, we observed an increase in the relative abundance of the hyperphosphorylated Orb isoforms in the three *cup* mutants. In wild type ovaries, the rapidly migrating lower isoform is always more abundant than the upper isoform (Fig. 8 and [19]) and the average ratio of upper to lower isoforms is 0.77. This ratio is approximately the same for extracts of ovaries containing all stages, and extracts prepared from young females that contain only pre-vitellogenic stages. In *cup* ovaries, we find that there is a consistent increase in the relative level of the upper isoform. As illustrated in Fig. 8, the average ratio of upper (hyperphosphorylated) to lower (hypophosphorylated) isoforms in *cup^8^* is 1.7. The average for *cup^3^* in three experiments was 1.2, while for *cup^1355^* it was 1.1. Though not as dramatic, we also observed an increase in the ratio of upper to lower isoforms when *HD19G orb^343^/+*females are *trans*-heterozygous for these three *cup* alleles.

## Discussion

Previous studies have shown that an autoregulatory mechanism is required to promote the accumulation of Orb protein in the oocyte of developing egg chambers [10]. Orb has two key activities in this autoregulatory loop. First, it functions in the localization of *orb* mRNA within the oocyte by anchoring the message to the oocyte cortex. Second, once *orb* mRNA is localized to the cortex, Orb promotes its on-site translation. In order to ensure that Orb protein accumulates at the oocyte cortex rather than elsewhere in the oocyte or in the nurse cells, it is block the activation of the autoregulatory loop until *orb* mRNA is properly localized. This means that there must be mechanisms in place to prevent not only the premature translation of *orb* mRNA while it is being transported from its site of synthesis in the nurse cells to oocyte cortex but also to block the precocious activation of the autoregulatory loop.

One factor that is known to be important in repressing the translation of *orb* mRNA is the *Drosophila* fragile X protein dFMR1 [11]. In *dfmr1* mutants, the translation of *orb* mRNA is upregulated and markedly higher levels of Orb are observed in mutant ovaries. However, while the total amount of Orb protein is elevated, its overall distribution in the egg chamber closely resembles that seen in wild type; the protein is concentrated in the oocyte, with only low levels in nurse cells. Moreover, although abnormal egg chambers are observed in *dfmr1* ovaries, they are relatively infrequent, and even in these chambers Orb concentrates in the presumptive oocyte. These findings suggest that dFMR1 functions as a general repressor of *orb* mRNA translation, but is not directly involved in preventing the precocious or inappropriate activation of the *orb* positive autoregulatory loop.

The studies reported here indicate that the *cup* gene also negatively regulates *orb* mRNA translation; however, it would appear to play a much more central role in controlling the *orb* positive autoregulatory loop than *dfmr1*. Several lines of evidence support this idea. The first evidence originates from genetic interactions studies between *cup* and *orb*. As would be predicted for a negative regulator, *cup* mutations dominantly suppress the DV polarity defects of eggs produced by females that have reduced *orb* activity. The second evidence is the pattern of Orb protein accumulation in *cup* mutant ovaries. In both of the strong *cup* alleles examined, Orb is precociously expressed in the nurse cells and high levels of Orb protein accumulate throughout much of the chamber. While Orb upregulation in *dfmr1* does not alter the relative partitioning of the protein into the nurse cell and oocyte compartments, this is not the case for the two Class I *cup* alleles. In fact, because high levels of Orb accumulate in multiple cells in the mutant chambers, it is often difficult to determine which cell corresponds to the oocyte based on the partitioning of the Orb protein. While the pattern of Orb accumulation in the Class III allele, *cup^1355^*, is relatively normal up till stage 5 or 6, *orb* mRNA appears to be precociously translated in stage 6–7 and older chambers and we detected elevated levels of Orb in the nurse cells. Significantly, studies in other laboratories have shown that like *orb*, *osk* mRNA is precociously translated in *cup* mutant ovaries [14]–[17]. Thirdly, the overall level of Orb protein is elevated compared to wild type in the two weaker *cup* alleles that were examined. While Orb levels are unexpectedly reduced in the strongest allele, *cup^8^*, our confocal analysis indicates that this is likely due to the fact Orb disappears from the mutant chambers as they age. Importantly, in the germarium, and in very young chambers, Orb levels (as detected by antibody staining) in *cup^8^* appear to be a good deal higher than in wild type. Fourth, while Orb protein levels are elevated, *orb* mRNAs levels are reduced in *cup* mutants compared to wild type. Since a similar reduction is observed for *osk* mRNA, it would appear that *cup* activity is needed either directly or indirectly to ensure the stability of at least two localized mRNAs. These unexpected effects of *cup* on mRNA stability would be consistent with recent studies in tissue culture cells, which have shown that tethering Cup protein to a reporter mRNAs stabilizes the mRNA even as it represses translation [22]. Finally, the idea that the *orb* autoregulatory loop is inappropriately activated is further supported by the finding that there is a shift in the *orb* mRNA poly(A) profile towards longer poly(A) tails in *cup* mutants.

The abnormal pattern of Orb protein accumulation in the two strong *cup* alleles also suggests that there may be problems in the specification or maintenance of oocyte identity. This possibility is supported by the fact that two other oocyte markers, Encore and *orb* mRNA, are also not properly localized. However, since we do see chambers in which these oocyte markers appear to be more highly concentrated in the presumptive oocyte, it seems likely that the loss of *cup* activity does not disrupt the initial specification of the oocyte, but rather affects the maintenance of oocyte identity. In this context, it is interesting to note that *orb* activity is required for the proper specification of the oocyte, and in strong *orb* mutants, oocyte identity is not properly established or maintained. Perhaps the presence of excessive amounts of Orb in the nurse cells of these *cup* mutant chambers might also compromise oocyte identity, but by a different mechanism. Instead of failing to express key oocyte determinants as in *orb* mutants, Orb would promote the premature expression of these determinants in the nurse cells.

An important question is why the *orb* autoregulatory loop becomes precociously activated in *cup* mutants? One factor that likely contributes to the premature activation of the *orb* autoregulatory loop is a failure to deadenylate *orb* mRNAs. Recent studies by Igreja and Izaurralde [22] have shown that one of the regulatory functions of the Cup protein is to recruit components of the deadenylation machinery and promote the deadenylation of its target mRNAs. Assuming that *cup* has a similar activity in the female germline, then it should directly antagonize Orb, which is thought to activate translation by promoting polyadenylation. A prediction of this model is that *orb* poly(A) tail lengths should increase when *cup* activity is reduced. Indeed, this is the case; we found that the poly(A) tails of *orb* mRNAs in *cup* mutants are elongated compared to wild type.

While an increase in poly(A) tail length would be expected to generally increase *orb* mRNA translation efficiency, a simple change in translational efficiency would not fully account for the effects of *cup* mutations. In particular, one might expect to observe a phenotype similar to that seen in *dfmr1* mutants where Orb protein levels are increased proportionally in both the nurse cells and the oocyte. However, in *cup* mutants there is a disproportionate increase in Orb protein levels in the nurse cells. This difference suggests that the *orb* autoregulatory loop is also precociously activated in nurse cells. In previous studies, we found that like the CPEB protein in Xenopus oocytes [27], Orb activity is modulated by phosphorylation [19]. We also found that phosphorylation is dependent upon CK2 activity. When *ck2* activity is partially compromised, there is a marked shift in the phosphoisoform profile towards the hypophosphorylated isoforms. Accompanying this shift in phosphoisoform profile, *orb* functions in DV polarity and *orb* autoregulation are disrupted. The results described here indicate that a shift in the opposite direction, *i.e.* towards the hyperphosphorylated isoforms occurs in *cup* mutant ovaries. Based on our previous work, such a shift in the phosphoisoform profile would be expected to upregulate *orb* activity. This change in phosphorylation status, like a failure in deadenylation, would be expected to contribute to the overexpression of Orb in *cup* mutant egg chambers. If this change in phosphorylation status were to occur in the nurse cells, it could lead to the precocious activation of the *orb* autoregulatory loop in the nurse cells and a disproportionate increase in the amount of Orb protein in these cells. Similarly, it could explain why the *orb* target *osk* mRNA is prematurely translated in *cup* mutants [14]–[17].

Since *cup* is a translational repressor, a plausible idea is that it indirectly downregulates phosphorylation by repressing the synthesis of CK2, some other kinase or the factors that control kinase activity. Alternatively (or in addition), the presence of Cup protein in Orb complexes could impede hyperphosphorylation. Consistent with this expectation, we were unable to detect Cup in Western blots of extracts from the two strongest alleles, *cup^8^* and *cup^3^*. *cup^1355^* is thought to encode a wild type protein and Cup-Orb complexes can be detected in this mutant (data not shown); however, as reported by Keyes and Spradling [13], Cup is expressed at a greatly reduced level (especially at later stages where effects on Orb expression are greatest) and it seems possible that Cup would be absent from many Orb complexes in this mutant.

While a model in which Cup inhibits phosphorylation (either directly or indirectly) would help explain why the levels of the hyperphosphorylated Orb isoforms increase in *cup* mutant ovaries, it leaves open the question of what happens in wild type ovaries. Since Cup promotes deadenylation, it should directly antagonize Orb activation of translation as long as the Cup protein remains associated with Orb containing mRNPs. Thus, a simple expectation is that Cup would dissociate from Orb mRNP complexes when Orb is hyperphosphorylated and activates translation. However, this does not seem to be the case as we find that Cup is associated with both hypo- (inactive) and hyper- (active) phosphorylated Orb (Fig. 1). Also, like Orb, Cup co-sediments with polysomes (unpublished data). If Cup remains associated with Orb mRNP complexes even after activation of translation, then there must be mechanisms that attenuate Cup dependent deadenylation and translational repression. One likely mechanism is that Cup-Orb mRNPs are re-organized upon activation and factors that facilitate Cup repressive activities are displaced. One such factor would be the Bruno repressor, which helps recruit Cup to target mRNAs. Bruno binds to many of the *orb* and *cup* regulatory targets including *orb* mRNA and like Cup it is thought to associate with newly synthesized mRNAs and repress translation while they are being transported into the oocyte [8]–[10], [15], [28], [29]. Once the mRNAs are transported into the oocyte, Bruno complexes containing Orb mRNA targets are reorganized and Bruno is displaced [19], [30]. It seems possible that factors like Not4 and Bicaudal-C, which function in deadenylation or translational repression, could also be displaced from the Cup-Orb complexes when Orb is activated. In addition to the reorganization of Cup-Orb mRNPs, it is possible that modifications that accompany translational activation, such as Orb phosphorylation help abrogate the repressive activities of the Cup protein.

## Materials and Methods

### *Drosophila* stocks

*HD19G orb^343^/Tm3Ser* flies were described in [10]. *cup* alleles used in this study were *cup^1^*, *cup^3^*, *cup^6^*, *cup^8^* and *cup^1355^*. *cup^1355^* was recovered in a P-element mutagenesis screen [31]; *cup^1^*, *cup^3^*, *cup^6^* and *cup^8^* were EMS-induced alleles recovered by Schüpbach and Wieschaus [12]. *cup^Δ212^* lacks the cup-eIF4E interaction domain and is described by Nakamura *et al.*, [15].

### Co-immunoprecipitation

Ovaries of 2–3 days old were fed on yeast overnight and dissected in 1× PBS. Ovary extract was prepared by homogenizing ovaries in Co-IP buffer (20 mM Hepes pH 7.5, 150 mM NaCl, 2.5 mM MgCl_2_, 250 mM sucrose, 0.05% NP40, 0.5% Triton-X, 1 mM PMSF, 1 µg/ml protease inhibitor cocktail, 1 mM dithiothreitol, 1 mM NaF, 40 µM NaVO3, 40 µM Na3VO4 and 500 µg of RNase A). The homogenate was centrifuged and the supernatant mixed with Orb antibody-coupled protein A/G beads (Calbiochem), and incubated at room temperature for 2 hours. The beads were washed with Co-IP buffer and the proteins that were immunoprecipitated analyzed by Western blots. The amount of ovary extract which was loaded in one control lane is approximately 10% of the amount of ovary extract that was used in the immunoprecipitation reactions loaded in one experimental lane.

### Western analysis

Protein samples were ran on ∼10% SDS-polyacrylamide gels and transferred onto PVDF membranes (Millipore). Blots were blocked in 5% milk/1× TBST for 2 hours, then incubated in primary antibodies (1∶30 anti-Orb 6H4 and 4H8; 1∶1000 anti-Cup [13], [16]; 1∶2500 anti-α-tubulin antibodies (Sigma)). Following rinsing, blots were incubated with secondary peroxidase-conjugated antibodies at 1∶1000 (Jackson ImmunoResearch Laboratories). Proteins were detected using chemiluminescence (ECL plus kit, Amersham Pharmacia Biotech). Quantification of protein levels were performed using ImageJ (NIH).

### Immunofluorescent staining

Ovaries were dissected and fixed in freshly made 4% paraformaldehyde. After rinsing, the ovaries were blocked in 10 mg/ml BSA/PBSTT. The samples were incubated in primary antibodies (1∶30 anti-Orb 6H4 and 4H8 in 1 mg/ml BSA/PBSTT) overnight at 4°C, then rinsed. Incubation in secondary antibodies (isotype-specific Alexa Fluor 568 goat anti-mouse IgG_2a_ and Alexa Fluor 488 goat anti-mouse IgG_1_ (Molecular Probes, Inc.) against 6H4 and 4H8, respectively) was performed at room temperature for approximately 2 hours. Microscopy was performed on a Perkin Elmer confocal microscope.

### *In situ* hybridization

Ovaries were dissected and fixed in 4% paraformaldehyde, then washed in PBSTT for 10 minutes twice. The fixed ovaries were incubated in 50 µg/ml of Proteinase K for 4 minutes. The reaction was stopped by incubation in 2 mg/ml glycine for 2 minutes. The ovaries were then washed in PBSTT for 5 minutes then re-fixed in 4% paraformaldehyde for 20 minutes. Following a wash in PBSTT, the ovaries were incubated in 1∶1 hybridization buffer∶PBSTT for 10 minutes at room temperature and hybridization buffer for another 10 minutes. Pre-hybridization was performed at 55°C for one hour. The probe was denatured at 80°C for 5 minutes and diluted to 1∶100. Hybridization with the probe was performed at 55°C overnight. After hybridization, the ovaries were equilibrated back to PBSTT by incubation in decreasing concentrations of hybridization buffer in PBSTT. The ovaries were blocked in 1% BSA/PBSTT for 30 minutes then incubated in 1∶5000 α-DIG for 90 minutes at room temperature, then rinsed in PBSTT and washed in alkaline wash. The ovaries were developed in 2% NBT/BCIP in alkaline wash.

### Quantitative real-time PCR

Approximately ten pairs of ovaries were hand-dissected for each experiment. Previtellogenic stages were separated from the post-vitellogenic stages; only the previtellogenic stages were used in these experiments and the post-vitellogenic stages were discarded. Total RNA was isolated using Trizol and reverse-transcribed. Quantitative real-time PCR was performed using primers specific for *orb* and *osk*. To normalize the amount of mRNA in the ovaries, *actin* specific primers were used as a control and as a proxy of relative total amounts of mRNA in the wild type and *cup* ovaries. The relative total amount of mRNA in the *cup* ovaries were calculated as a ratio of the *actin* mRNA in *cup* mutants to the amount of *actin* mRNA in the wild type ovaries. The relative amount of *orb* and *osk* mRNA in the *cup* ovaries is calculated as a ratio of amounts of *orb* and *osk* mRNA to the amounts of *orb* and *osk* mRNA in the wild type ovaries. To compare the relative amounts of *orb* and *osk* mRNA in the mutants relative to the wild type, the ratio of the relative amounts of *orb* and *osk* mRNA in *cup* mutants to the relative total amount of *actin* mRNA is calculated.

## Supporting Information

Figure S1 ***cup*** **mutants have defects in oocyte determination.** Green: Encore. Red: Orb. WT: In wild type ovaries, Encore and Orb are concentrated in the presumptive oocyte in newly formed 16 cell cysts in the germarium. During early pre-vitellogenic stages the localization pattern is further refined so that only the oocyte has high levels of these two proteins. In the germarium of *cup^8^* ovaries, Encore and Orb are distributed in most of the cells in the 16-cell cyst (see arrow). The two proteins are also not properly concentrated into the presumptive oocyte in many older mutant egg chambers. Instead, several cells have high levels of Encore and Orb (see arrowheads).
(TIF)

Figure S2 **Poly(A) tails are elongated in** ***cup^1355^*** **.** Poly(A) tails of *orb* mRNA from wild type and *cup^1355^* (as indicated) were analyzed using the anchored poly(A)-tail assay [24]. Anchor primers for reverse transcription and amplification from the poly(A) tail were as described in [24], while the nested *orb* specific primers (F2 and F4) were derived from the *orb* 3′ UTR. The amplification products were analyzed on an agarose gel and visualized with ethidium bromide.
(PDF)

Table S1 ***cup*** **negatively regulates** ***orb*** **and suppresses the ventralization phenotype of** ***HD19Gorb^343^*** **.** Females that were trans-heterozygous for *HD19Gorb^343^* and five different alleles of *cup* mutants were generated by crossing *HD19G orb^343^/TM3Ser* females (n = 10 in each cross) with *cup/CyO* males. Independent crosses were set up and scored at 18°C and 25°C. The number of embryos scored at each temperature is shown in the table. While approximately 20–30% of embryos laid by *HD19G orb^343^/+* females were ventralized, suppression of this phenotype was seen in trans-heterozygotes of all five alleles. Approximately 2.5–7.3% of embryos laid by *cup* mutant transheterozygotes were ventralized at 18°C. Suppression was weaker but still very obvious and statistically significant at 25°C, where 8.6–20.4% of embryos were ventralized.
(DOC)

## References

[1] Bilger A, Fox CA, Wahle E, Wickens M (1994). Nuclear polyadenylation factors recognize cytoplasmic polyadenylation elements.. Genes Dev.

[2] Hake LE, Richter JD (1994). CPEB is a specificity factor that mediates cytoplasmic polyadenylation during Xenopus oocyte maturation.. Cell.

[3] Lantz V, Ambrosio L, Schedl P (1992). The Drosophila orb gene is predicted to encode sex-specific germline RNA-binding proteins and has localized transcripts in ovaries and early embryos.. Development.

[4] Christerson LB, McKearin DM (1994). orb is required for anteroposterior and dorsoventral patterning during *Drosophila* oogenesis.. Genes Dev.

[5] Lantz V, Chang JS, Horabin JI, Bopp D, Schedl P (1994). The *Drosophila* orb RNA-binding protein is required for the formation of the egg chamber and establishment of polarity.. Genes Dev.

[6] Huynh JR, St Johnston D (2000). The role of BicD, Egl, Orb and the microtubules in the restriction of meiosis to the Drosophila oocyte.. Development.

[7] Chang JS, Tan L, Wolf MR, Schedl P (2001). Functioning of the Drosophila orb gene in gurken mRNA localization and translation.. Development.

[8] Chang JS, Tan L, Schedl P (1999). The *Drosophila* CPEB homolog, orb, is required for oskar protein expression in oocytes.. Dev Biol.

[9] Castagnetti S, Ephrussi A (2003). Orb and a long poly(A) tail are required for efficient oskar translation at the posterior pole of the *Drosophila* oocyte.. Development.

[10] Tan L, Chang JS, Costa A, Schedl P (2001). An autoregulatory feedback loop directs the localized expression of the *Drosophila* CPEB protein Orb in the developing oocyte.. Development.

[11] Costa A, Wang Y, Dockendorff TC, Erdjument-Bromage H, Tempst P (2005). The *Drosophila* fragile X protein functions as a negative regulator in the orb autoregulatory pathway.. Dev Cell.

[12] Schüpbach T, Wieschaus E (1986). Germline autonomy of maternal-effect mutations altering the embryonic body pattern of Drosophila.. Dev Biol.

[13] Keyes LN, Spradling AC (1997). The *Drosophila* gene fs(2)cup interacts with otu to define a cytoplasmic pathway required for the structure and function of germ-line chromosomes.. Development.

[14] Wilhelm JE, Hilton M, Amos Q, Henzel WJ (2003). Cup is an eIF4E binding protein required for both the translational repression of oskar and the recruitment of Barentsz.. J Cell Biol.

[15] Nakamura A, Sato K, Hanyu-Nakamura K (2004). *Drosophila* Cup is an eIF4E binding protein that associates with Bruno and regulates oskar mRNA translation in oogenesis.. Dev Cell.

[16] Nelson MR, Leidal AM, Smibert CA (2004). *Drosophila* Cup is an eIF4E-binding protein that functions in Smaug-mediated translational repression.. EMBO J.

[17] Zappavigna V, Piccioni F, Villaescusa JC, Verrotti AC (2004). Cup is a nucleocytoplasmic shuttling protein that interacts with the eukaryotic translation initiation factor 4E to modulate *Drosophila* ovary development.. Proc Natl Acad Sci USA.

[18] Chekulaeva M, Hentze MW, Ephrussi A (2006). Bruno acts as a dual repressor of oskar translation, promoting mRNA oligomerization and formation of silencing particles.. Cell.

[19] Wong LC, Costa A, McLeod I, Sarkeshik A, Yates J (2011). The functioning of the *Drosophila* CPEB protein Orb is regulated by phosphorylation and requires Casein kinase 2 activity. PLoS ONE.. 2011;.

[20] Van Buskirk C, Hawkins NC, Schüpbach T (2000). Encore is a member of a novel family of proteins and affects multiple processes in *Drosophila* oogenesis.. Development.

[21] Radford HE, Meijer HA, de Moor CH (2008). Translational control by cytoplasmic polyadenylation in Xenopus oocytes.. Biochim Biophys Acta.

[22] Igreja C, Izaurralde E (2011). Cup promotes deadenylation and inhibits decapping of mRNA targets.. Genes Dev.

[23] Sallés FJ, Lieberfarb ME, Wreden C, Gergen JP, Strickland S (1994). Coordinate initiation of Drosophila development by regulated polyadenylation of maternal messenger RNAs.. Science.

[24] Salles FJ, Richards WG, Strickland S (1999). Assaying the polyadenylation state of mRNAs.. Methods.

[25] Dickson KS, Thompson SR, Gray NK, Wickens M (2001). Poly(A) polymerase and the regulation of cytoplasmic polyadenylation.. J Biol Chem.

[26] Benoit P, Papin C, Kwak JE, Wickens M, Simonelig M (2008). PAP- and GLD-2-type poly(A) polymerases are required sequentially in cytoplasmic polyadenylation and oogenesis in Drosophila.. Development.

[27] Mendez R, Murthy KG, Ryan K, Manley JL, Richter JD (2000). Phosphorylation of CPEB by Eg2 mediates the recruitment of CPSF into an active cytoplasmic polyadenylation complex.. Mol Cell.

[28] Webster PJ, Liang L, Berg CA, Lasko P, Macdonald PM (1997). Translational repressor bruno plays multiple roles in development and is widely conserved.. Genes Dev.

[29] Kim-Ha J, Kerr K, Macdonald PM (1995). Translational regulation of oskar mRNA by Bruno, an ovarian RNA-binding protein, is essential.. Cell.

[30] Snee MJ, MacDonald PM (2009). Dynamic organization and plasticity of sponge bodies.. Dev Dyn.

[31] Karpen GH, Spradling AC (1992). Analysis of subtelomeric heterochromatin in the *Drosophila* minichromosome Dp1187 by single P element insertional mutagenesis.. Genetics.

