# Supplementary figures and images for: Cup Blocks the Precocious Activation of the Orb Autoregulatory Loop
Source: PLoS One. 2011 Dec 2;6(12):e28261. doi: 10.1371/journal.pone.0028261 (PMC3229553; doi:10.1371/journal.pone.0028261)

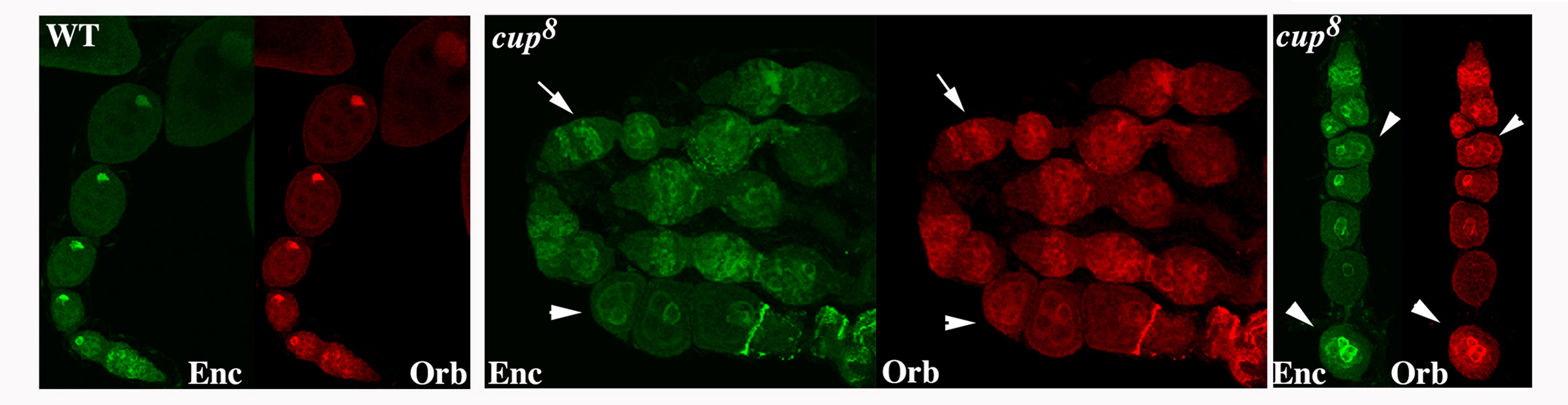

Supplement: Figure S1 — cup mutants have defects in oocyte determination. Green: Encore. Red: Orb. WT: In wild type ovaries, Encore and Orb are concentrated in the presumptive oocyte in newly formed 16 cell cysts in the germarium. During early pre-vitellogenic stages the localization pattern is further refined so that only the oocyte has high levels of these two proteins. In the germarium of cup8 ovaries, Encore and Orb are distributed in most of the cells in the 16-cell cyst (see arrow). The two proteins are also not properly concentrated into the presumptive oocyte in many older mutant egg chambers. Instead, several cells have high levels of Encore and Orb (see arrowheads). (TIF) [file pone.0028261.s001.tif]

*cup*<sup>1355</sup> WT

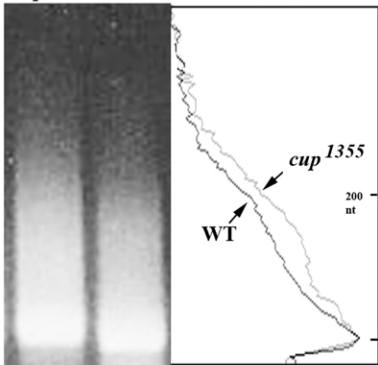

Supplement: Figure S2 — Poly(A) tails are elongated in cup1355 . Poly(A) tails of orb mRNA from wild type and cup1355 (as indicated) were analyzed using the anchored poly(A)-tail assay [24]. Anchor primers for reverse transcription and amplification from the poly(A) tail were as described in [24], while the nested orb specific primers (F2 and F4) were derived from the orb 3′ UTR. The amplification products were analyzed on an agarose gel and visualized with ethidium bromide. (PDF) [file pone.0028261.s002.pdf]
