# Supplementary material for: Cup Blocks the Precocious Activation of the Orb Autoregulatory Loop
Source: PLoS One. 2011 Dec 2;6(12):e28261. doi: 10.1371/journal.pone.0028261 (PMC3229553; doi:10.1371/journal.pone.0028261)
Supplement: Table S1 — cup negatively regulates orb and suppresses the ventralization phenotype of HD19Gorb343 . Females that were trans-heterozygous for HD19Gorb343 and five different alleles of cup mutants were generated by crossing HD19G orb343/TM3Ser females (n = 10 in each cross) with cup/CyO males. Independent crosses were set up and scored at 18°C and 25°C. The number of embryos scored at each temperature is shown in the table. While approximately 20–30% of embryos laid by HD19G orb343/+ females were ventralized, suppression of this phenotype was seen in trans-heterozygotes of all five alleles. Approximately 2.5–7.3% of embryos laid by cup mutant transheterozygotes were ventralized at 18°C. Suppression was weaker but still very obvious and statistically significant at 25°C, where 8.6–20.4% of embryos were ventralized. (DOC) [file pone.0028261.s003.doc]

|  | 18ºC | | |
| --- | --- | --- | --- |
|  | ventralized/total | % ventralized | p-value |
| *HD19G orb^343^/+* | 327/1139 | 28.71% |  |
| *cup^1355^ /+ ; HD19G orb^343^/+* | 28/848 | 3.31% | 1.72E-28 |
| *cup^1^ /+ ; HD19G orb^343^/+* | 44/1033 | 4.26% | 3.03E-141 |
| *cup^3^ /+ ; HD19G orb^343^/+* | 44/1117 | 3.94% | 1.73E-29 |
| *cup^6^ /+ ; HD19G orb^343^/+* | 32/1309 | 2.44% | 7.63E-36 |
| *cup^8^ /+ ; HD19G orb^343^/+* | 91/1243 | 7.32% | 7.66E-14 |

|  |  |  |  |
| --- | --- | --- | --- |
|  | 25ºC | | |
|  | ventralized/total | % ventralized | p-value |
| *HD19G orb^343^/+* | 314/1463 | 21.46% |  |
| *cup^1355^ /+ ; HD19G orb^343^/+* | 87/797 | 10.92% | 4.53E-07 |
| *cup^1^ /+ ; HD19G orb^343^/+* | 103/1198 | 8.60% | 5.43E-118 |
| *cup^3^ /+ ; HD19G orb^343^/+* | 245/1203 | 20.37% | 0.608012094 |
| *cup^6^ /+ ; HD19G orb^343^/+* | 168/1627 | 10.33% | 4.58E-09 |
| *cup^8^ /+ ; HD19G orb^343^/+* | 74/710 | 10.42% | 0.000309325 |
|  |  |  |  |

**Supplemental table 1: *cup* interacts genetically with *orb* and suppresses the ventralization phenotype of *HD19Gorb^343^.***
